# Comments on the introduction of the paper "Overview of the biochemical and genetic processes in malignant mesothelioma"

**DOI:** 10.1590/S1806-37132014000500017

**Published:** 2014

**Authors:** Algranti Eduardo

**Affiliations:** Department of Medicine, Fundação Jorge Duprat Figueiredo de Segurança e Medicina do Trabalho – FUNDACENTRO, Jorge Duprat Figueiredo Foundation for Occupational Safety and Medicine – São Paulo, Brazil

## To the Editor:

I read with interest the article "Overview of the biochemical and genetic processes in malignant mesothelioma", by Assis and Isoldi. ^(^ 1 ^)^ It is a comprehensive literature review of the mechanisms of tumorigenesis in malignant mesothelioma (MM) and includes studies conducted by the authors themselves. The topic is relevant because there is evidence of a gradual increase in the incidence of MM in Brazil, particularly in southeastern Brazil,^(^ 2 ^)^ despite strong evidence of underreporting. Although the prognosis of MM is extremely poor, MM is preventable, given that it is of occupational or environmental origin in the vast majority of cases. 

Although the title of the review article describes the scope of the study, the two-page introduction addresses general issues regarding MM and is the reason why the present letter was written. 

First, I would like to comment on simian virus 40 (SV40). In the abstract of their article,^(^ 1 ^)^ the authors state that "the development of MM is strongly correlated with exposure to asbestos and erionite, as well as to simian virus 40". At the end of the sixth paragraph of the introduction, the statement is repeated, albeit in other words, being accompanied by four references. 

In the 1960s, some batches of polio vaccine were contaminated with SV40. Since then, there has been interest in studying the consequences of this accidental inoculation in individuals who received SV40-contaminated vaccines. More than 20 years ago, it was hypothesized that SV40 played a role in the genesis of MM because of its potential to cause mesothelioma in laboratory animals.^(^ 3 ^)^ Subsequent studies demonstrated the role of SV40 in a significant proportion of MM cases.^(^ 4 ^,^ 5 ^)^ However, epidemiological studies of populations exposed to contaminated vaccines showed no increased incidence of cancer, including MM.^(^ 6 ^)^ Two studies used multiple techniques for detecting SV40 in MM and normal kidney tissue, avoiding cross-reactions with other polyomaviruses, and neither showed the presence of SV40 in the samples analyzed.^(^ 7 ^,^ 8 ^)^ In contrast, in one study, indirect ELISA demonstrated a higher prevalence of serum antibodies to SV40 in patients with MM than in healthy controls.^(^ 9 ^)^

Recently, the International Agency for Research on Cancer (IARC) published a comprehensive review of the carcinogenicity of polyomaviruses, concluding that there is inadequate evidence in humans for the carcinogenicity of SV40, which was therefore classified as a Group 3 carcinogen,^(^ 10 ^)^ meaning that SV40 is not classifiable as to its carcinogenicity to humans. In the aforementioned review,^(^ 10 ^)^ the reasons for conflicting results across studies are discussed. On the basis of the available evidence, it is incorrect to state that MM is strongly correlated with SV40. What is even worse is that the statement as it is misleads readers to believe that SV40 and asbestos have the same potential to cause MM. 

Second, I would like to comment on the statement that "there is considerable debate regarding the role of chrysotile asbestos in the genesis of MM". The authors continue by stating that "there are reports that chrysotile asbestos cannot cause MM in humans", without providing any reference to support this assertion. That is not the consensus! According to the IARC, chrysotile is a Group 1 carcinogen to human mesothelial cells.^(^ 11 ^)^ The potential of chrysotile asbestos to cause MM has been reported to be one order of magnitude lower than that of amphibole asbestos.^(^ 12 ^)^

Assis and Isoldi are experts in the biochemical and genetic processes associated with MM, as evidenced by the references to studies conducted by the group. Their review of the genes and biochemical pathways involved in MM is excellent; however, the introduction of the article mars their work by including poorly substantiated or incorrect statements regarding the potency of MM-inducing agents and the causal relationships between human exposure to those agents and the development of MM (all of which have been widely discussed in the literature), presenting readers with recurrent misconceptions.
