# Supplementary material for: Comments on the introduction of the paper "Overview of the biochemical and genetic processes in malignant mesothelioma"
Source: J Bras Pneumol. 2014 Sep-Oct;40(5):584–5. doi: 10.1590/S1806-37132014000500017 (PMC4263342; doi:10.1590/S1806-37132014000500017)
Supplement: Translation

# Translation: Comentários sobre a introdução do artigo "Panorama dos processos bioquímicos e genéticos presentes no mesotelioma maligno"

**Authors:** Eduardo Algranti

**Affiliations:** Medicina Fundação Jorge Duprat Figueiredo de Segurança e Medicina do Trabalho – FUNDACENTRO – São Paulo (SP) Brasil

## Ao Editor:

Li com interesse o artigo de Assis e Isoldi intitulado *"*Panorama dos processos bioquímicos e genéticos presentes no mesotelioma maligno". ^(^ 1 ^)^ O texto traz uma revisão dos mecanismos na tumorigênese do mesotelioma maligno (MM), embasado numa ampla revisão bibliográfica, que inclui trabalhos originais dos próprios autores. O tema é relevante uma vez que há indicações de um aumento paulatino da incidência do MM no Brasil, particularmente na região Sudeste,^(^ 2 ^)^ mesmo com os fortes indícios de subnotificação e subregistro. É um câncer de péssimo prognóstico, evitável, uma vez que, na esmagadora maioria dos casos, tem uma causa ocupacional ou ambiental.

Embora o objeto do texto esteja definido no título da revisão, os autores fazem uma introdução de duas páginas envolvendo questões gerais sobre o MM, motivo dos comentários da presente carta.

O primeiro comentário refere-se ao vírus símio 40 (SV40). No resumo da referida publicação,^(1) ^os autores afirmam que "o desenvolvimento do MM é fortemente correlacionado com a exposição ao amianto e erionita, assim como ao vírus símio 40". Ao final do sexto parágrafo da introdução, o comentário é repetido com outra redação acompanhada de quatro referências bibliográficas.

Nos anos 60 do último século, alguns lotes de vacinas antipólio foram contaminados pelo SV40. Desde então, houve interesse no estudo das repercussões da inoculação acidental do SV40 em populações que receberam os lotes contaminados. Há mais de 20 anos levantou-se a hipótese de participação do SV40 na gênese do MM pelo seu potencial de causar mesoteliomas em animais de laboratório.^(^ 3 ^)^ Posteriormente, seguiram-se estudos que demonstraram a participação do SV40 em uma proporção importante de casos de MM.^(^ 4 ^,^ 5 ^)^ Entretanto, estudos epidemiológicos em populações expostas a vacinas contaminadas não demonstraram um aumento na incidência de câncer, incluindo-se o MM.^(^ 6 ^)^ Dois estudos, envolvendo múltiplas técnicas de detecção viral de SV40 em tecidos de MM e de rins normais, evitando-se reações cruzadas com outros poliomavírus, não demonstraram a presença do SV40 nas amostras analisadas.^(^ 7 ^,^ 8 ^)^ Em contrapartida, utilizando-se um método de ELISA indireto, demonstrou-se uma maior prevalência de anticorpos séricos contra o SV40 em pacientes portadores de MM.^(^ 9 ^)^

Recentemente, a *International Agency for Research on Cancer* (IARC) publicou uma ampla revisão sobre a carcinogenicidade dos poliomavírus, concluindo que as evidências do SV40 em humanos são inadequadas, sendo classificado como carcinógeno do Grupo 3,^(^ 10 ^)^ o que significa que o SV40 não é classificável como cancerígeno para humanos. Naquele texto,^(^ 10 ^)^ discutem-se as razões de resultados discordantes em estudos publicados. Frente às evidências disponíveis, afirmar-se que o MM é fortemente correlacionado com o SV40 é incorreto e, o que é ainda pior, da forma como está no texto, induz o leitor a equiparar o SV40 ao potencial indutor do asbesto.

O segundo comentário refere-se à menção dos autores sobre "um grande debate mundial" na participação do amianto do tipo crisotila na gênese do MM. Os autores prosseguem, afirmando que "há relatos de que o amianto crisotila não é capaz de causar MM em humanos", sem fornecerem qualquer referência que sustente tal assertiva. O consenso não é esse! A crisotila é considerada como cancerígena do Grupo 1 para o mesotélio pelo IARC.^(^ 11 ^)^ O que se admite é que o potencial cancerígeno da crisotila para o mesotélio é inferior aos do tipo anfibólio em uma ordem de grandeza.^(^ 12 ^)^

Os autores são especialistas no ponto focal do artigo. Prova disso são as referências de trabalhos produzidos pelo grupo. O texto sobre genes e vias bioquímicas é uma excelente revisão do tema; porém, a introdução tropeça ao utilizar afirmativas mal sustentadas ou incorretas sobre as relativas relações de força dos agentes indutores do MM e sobre as relações de causalidade da doença, fartamente discutidas e disponíveis na literatura científica, o que compromete a publicação ao introduzir conceitos recorrentemente errôneos ao leitor.
